# Adjunct diagnostic value of radiological findings in mucopolysaccharidosis type IVa-related thoracic spinal abnormalities: a pilot study

**DOI:** 10.1186/s13023-022-02449-9

**Published:** 2022-07-29

**Authors:** Ya-Ting Jan, Pei-Shan Tsai, Wen-Hui Huang, Shih-Chieh Huang, Yu-Peng Liu, She-Meng Cheng, Kun-Shuo Huang

**Affiliations:** 1 Department of Radiology, MacKay Memorial Hospital, No. 92, Sec. 2, Zhongshan N. Rd., Taipei City, 104 Taiwan; 2 Department of Radiology, MacKay Memorial Hospital, Hsinchu, 300 Taiwan; 3 Department of Biomedical Imaging and Radiological Sciences, National Yang Ming Chiao Tung University, Taipei, 112 Taiwan; 4 Department of Medicine, Mackay Medical College, New Taipei City, 252 Taiwan; 5 Nursing and Management, Mackay Junior College of Medicine, New Taipei City, 112 Taiwan

**Keywords:** Mucopolysaccharidosis, Spinal stenosis, Thoracic kyphosis, Thoracic vertebral body

## Abstract

**Background:**

In patients with mucopolysaccharidosis (MPS), systematic assessment and management of cervical instability, cervicomedullary and thoracolumbar junction spinal stenosis and spinal cord compression averts or arrests irreversible neurological damage, improving outcomes. However, few studies have assessed thoracic spinal involvement in MPS IVa patients. We aimed to evaluate thoracic spinal abnormalities in MPS IVa patients and identify associated image manifestations by CT and MRI study.

**Results:**

Data of patients diagnosed and/or treated for MPS IVa at MacKay Memorial Hospital from January 2010 to December 2020 were extracted from medical records and evaluated retrospectively. Computed tomography (CT), plain radiography and magnetic resonance imaging (MRI) findings of MPS IVa-related spinal abnormalities were reviewed. Spine CT and plain radiography findings of 12 patients (6 males and 6 females with median age 7.5 years, range 1–28 years) revealed two subtypes of spinal abnormalities: thoracic kyphosis apex around T2 (subtype 1, n = 8) and thoracic kyphosis apex around T5 (subtype 2, n = 4). Spine CT and plain radiography clearly identified various degrees of thoracic kyphosis with apex around T2 or T5 in MPS IVa patients. Square-shaped to mild central beaking in middle thoracic vertebral bodies was observed in subtype 1 patients, while greater degrees of central beaking in middle thoracic vertebral bodies was observed in subtype 2 patients.

**Conclusions:**

Spine CT findings clearly identify new radiological findings of thoracic kyphosis apex around T2 or T5 in MPS IVa patients. The degrees of central beaking at middle thoracic vertebral bodies may be a critical factor associated with different image presentations of thoracic kyphosis.

## Background

Mucopolysaccharidosis (MPS) belongs to a group of genetic disorders known as lysosomal storage diseases (LSDs). Deficiencies of specific enzymes in MPS disease interrupt the degradation of mucopolysaccharides (or glycosaminoglycans), leading to accumulation of these substances in cellular lysosomes. MPS disease includes 15 types, which are divided into 7 phenotypes according to the specific enzyme deficiency: MPS types I, II, III, IV, VI, VII, IX [1, 2]. All phenotypes have an autosomal recessive hereditary pattern except MPS type II, which has an X-linked recessive pattern [1–3]. MPS type IVa, or Morquio A syndrome, is caused by deficiency of N-acetylgalactosamine-6-sulfate sulphatase (a catalyzed enzyme that breaks down into two glycosaminoglycans [GAG], keratan sulphate [KS] and chondroitin 6-sulphate [C6S]), and consequent gradual accumulation of KS and C6S in several organs and tissues. Because KS and C6S are the main components of proteoglycans in cartilage and bone, MPS IVa presents primarily with skeletal dysplasia and short stature [4, 5]. Skeletal deformity is defined as multiple bone dysostosis occurring as the most common initial symptom [5, 6]. Visual, auditory, cardiovascular and respiratory systems are also compromised in this multisystem disease [4, 5, 7]. Although MPS IVa is generally not believed to affect neurocognitive function [5], a recent study suggests that subtle neurocognitive involvement may exist [8]. Symptom onset typically occurs before 1 year of age in patients with rapidly progressive disease, and usually occurs in the second decade of life in patients with slowly progressive disease [6]. In individuals with phenotypes of severe disease, paralysis from cervical myelopathy, respiratory insufficiency and cardiac abnormalities may all shorten the lifespan, with death typically occurring in the second or third decade [5, 6]. In contrast, MPS IVa attenuated patients may have normal or near-normal life expectancy [5, 6].

In general, spinal involvement in MPS IVa occurs at two distinct sites. Involvement of the cervical spine, particularly spinal instability and spinal cord compression at the C1-C2 level, is a nearly universal finding and predisposes patients to myelopathy, paralysis and sudden death [5]. Spinal cord compression due to thoracolumbar kyphosis is uncommon but can lead to insidious paraplegia with all of its devastating consequences [9]. Prevention of these complications requires early detection and prompt treatment of cervical instability, spinal stenosis and spinal cord compression. Spinal involvement is usually progressive, but neurological deficits may occur suddenly [4]. Neurological signs and symptoms may underestimate the severity of spinal cord involvement revealed by MRI [4, 10], emphasizing the importance of advanced imaging in managing spinal disease in MPS IVa.

Recently, Solanki et al. [11] reviewed the complex anatomy and pathology of the cervical atlantoaxial region, outlining spinal involvement in MPS IVa. Through systematic evaluation and management of spinal instability, cervical and thoracolumbar spinal stenosis and spinal cord compression, irreversible neurological damage can be avoided or prevented to improve patient outcomes [11]. The correlation between clinical and neurological findings and imaging studies is particularly important [11]. CT and plain radiography are useful imaging modalities for detecting vertebral bony abnormalities. Detailed visualization of multiple spinal defects makes MRI the most appropriate and useful imaging technique for assessing the degree of spinal stenosis and spinal cord compression [11, 12]. However, few studies have assessed thoracic spinal involvements in patients with MPS IVa. Therefore, this study aimed to assess the unique thoracic spinal computed tomography and MRI image manifestations in patients with MPS IVa.

## Methods

### Study design and sample

This retrospective study enrolled patients with MPS IVa who were diagnosed and/or treated at MacKay Memorial Hospital from January 2010 to December 2020. 26 cases of MPS IVa were collected during this study period, including 14 outpatients and 12 inpatients. Retrospective chart review of data from above patients was performed. The inclusion criteria were having qualified spinal CT or MRI and confirmation of a known diagnosis of MPS IVa by liquid chromatography/tandem mass spectrometry (LC-MS/MS) method for three urinary glycosaminoglycans (GAGs; dermatan sulfate [DS], heparan sulfate [HS], and keratan sulfate [KS]). Patients who did not undergo associated spinal CT or MRI radiological examination, as well as those with only postoperative images in their electronic medical records at our institute, were excluded. Finally, 12 MPS IVa patients were enrolled in this study.

### Ethical considerations

The study protocol was approved by the medical ethics committee of MacKay Memorial Hospital (21MMHIS335e). Due to the retrospective nature of this study, the ethics committee waived signed informed consent from included patients.

### MRI and CT assessment

All included patients received CT and/or MRI, as well as plain radiography to evaluate MPS IVa-related symptoms. CT was performed for these patients because of clinical symptoms such as respiratory insufficiency, cardiac or spinal problems using Somatom Definition Flash (Siemens Healthcare, Forchheim, Germany) or Somatom Definition AS (Siemens Healthcare, Forchheim, Germany) scanners. Subsequent reconstructed spinal CT images were obtained for image analysis using the bone window setting in the sagittal plane with 2 mm slice thickness. Spinal MRI was performed for clinical screening to assess the conditions around cervicomedullary and the thoracolumbar junction using a 1.5 T GE Signa Twinspee and Signa HDxt scanners (GE Healthcare, Waukesha, WI, USA). All spinal MRI studies comprised T1-weighted (T1WI), T2-weighted (T2WI) and short-tau inversion recovery (STIR) images in sagittal, and T1-weighted (T1WI), T2-weighted (T2WI) images in axial sections of 3 mm slice thickness. All patients were placed in the supine position on CT/MRI. Each imaging examination was reviewed by two radiologists both with 10 + years of experience.

### Data collection and assessment

Patients’ demographics (age at study enrollment and gender), clinical symptoms for CT/MRI examination, and records of whether enzyme replacement therapy (ERT) was administered were collected. Radiological findings including odontoid dysplasia and craniocervical junction abnormalities, thoracic and thoracolumbar kyphotic apex with associated spinal stenosis, central beaking of middle thoracic vertebral bodies, as well as the Cobb angle of thoracic kyphosis were evaluated and measured by CT or MRI images. Presence of dens hypoplasia or os odontoideum suggested odontoid dysplasia [11, 13]. Spinal stenosis was evaluated by the severity of obliterated CSF space around spinal cord and was assessed for presence of spinal cord compressive myelopathy [11, 14]. We further classified central beaking of middle thoracic vertebral bodies of our enrolled patients into 3 subgroups – (1) square-shaped vertebral body (“‒”, no significant central beaking), (2) square-shaped to mild central beaking (“+”, central beaking < 1/3 height of vertebral body), and (3) mild to moderate central beaking (“++”, central beaking between 1/3 and 2/3 height of vertebral body). The thoracic kyphotic angle was measured by the widely used gold standard method, the Cobb angle [15]. Lastly, to rule out the possibility of potential bias, the above images were reviewed independently by two senior radiologists. If there was a disagreement in the image findings, the two reviewers reached consensus through discussion.

## Results

### Patients’ baseline characteristics

During the study period, twelve MPS IVa patients (6 males and 6 females; 4 outpatients and 8 inpatients; median age 7.5 years, ranging 1–28 years of age) who were diagnosed and/or treated at our institute were enrolled. Six cases had both spinal CT/MRI images, four had only CT images, two had only MR images, and all patients had spinal plain radiography. Patients’ baseline demographic, clinical indications for CT/MRI study, as well as status of enzyme replacement therapy (ERT) are listed in Table 1.

**Table 1:** Demographic characteristics and thoracic spinal radiological findings of mucopolysaccharidosis type IVa patients

	Patient 1	Patient 2	Patient 3	Patient 4	Patient 5	Patient 6	Patient 7	Patient 8	Patient 9	Patient 10	Patient 11	Patient 12
Age (y/o) at study enrollment	15	14	9	2	6	7	5	15	26	1	1	28
Gender	Female	Female	Male	Female	Female	Female	Male	Female	Male	Male	Male	Male
Clinical indications for CT/MRI exam	Obstructive airways	Obstructive airways and craniocervical junction abnormalities	Obstructive airways, heart disease and craniocervical junction abnormalities	Craniocervical junction abnormalities	Obstructive airways	Craniocervical junction abnormalities	Obstructive airways, heart disease and craniocervical junction abnormalities	Obstructive airways and craniocervical junction abnormalities	Obstructive airways and heart disease	Craniocervical junction abnormalities	Thoracic kyphosis	Obstructive airways, heart disease and craniocervical junction abnormalities
CT	√	√	√	√	√	√	√	√	√			√
MRI		√	√			√	√	√		√	√	√
ERT						√	√	√	√	√	√	√
Subtype of thoracic kyphosis	1	1	1	1	2	2	2	1	1	1	2	1
Thoracic kyphosis apex	T2	T1/2	T2	T2	T5	T5	T4/5	T2	T2	T2	T5	T2
Cobb angle of thoracic kyphosis	0 –10 degrees	10 –20 degrees	10 –20 degrees	10 –20 degrees	20 – 30 degrees	30 –40 degrees	20 –30 degrees	10 –20 degrees	20 –30 degrees	10 –20 degrees	20 –30 degrees	10 –20 degrees
Central beaking of middle thoracic vertebral body	‒	+	+	+	++	++	++	‒	+	‒	++	+
Thoracolumbar kyphosis apex	L1	T11	T12	T12	L2	T11	L1	L1	T12	L1	L2	T11

*CT* Computed tomography;* MRI* Magnetic resonance imaging;* ERT* Enzyme replacement therapy;* T* Thoracic;* L* Lumbar; √, yes; “‒” indicates square-shaped vertebral body (no significant central beaking); “+” indicates square-shaped to mild central beaking (central beaking < 1/3 height of vertebral body); “++” indicates mild to moderate central beaking (central beaking between 1/3 and 2/3 height of vertebral body)

### Spine CT and plain radiographs

**Fig. 1:**
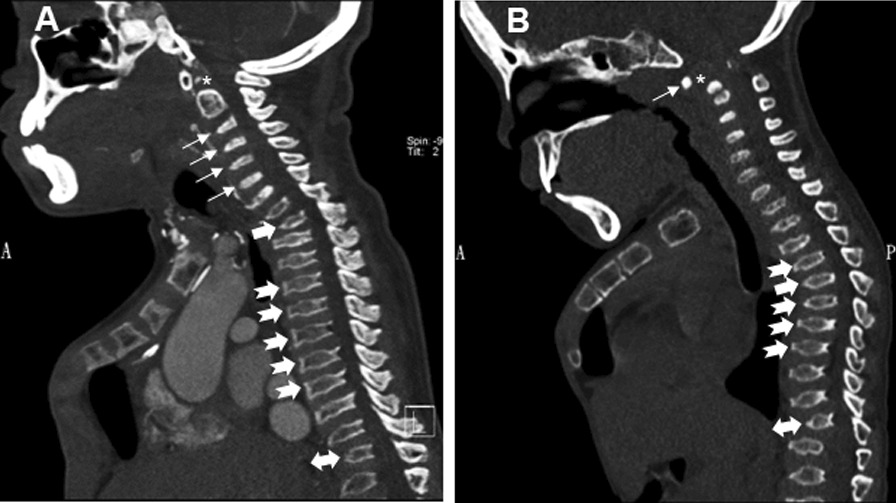
Spinal CT manifestations in MPS IVa patients. Sagittal CT images grouped into subtypes 1 and 2 according to different levels of thoracic kyphosis apex. **A** MPS type IVa in a 26-year-old man (subtype 1). Sagittal reformatted spinal CT images show cervicothoracic kyphosis with apex around level of T2 (solid arrow), accompanied by square-shaped to mild central beaking of middle thoracic vertebral bodies (notched arrow). Mild thoracolumbar kyphosis with apex around level of T12 (double arrow), anterior central beaking of cervical (arrow) and visible lumbar vertebral bodies, and odontoid dysplasia (asterisk) are noted as well. **B** MPS type IVa in a 7-year-old girl (subtype 2). Sagittal reformatted CT images of the spine show middle thoracic kyphosis with apex around T5 (solid arrow), along with greater degrees of anterior central beaked thoracic vertebral bodies (notched arrow). Common spinal involvement of odontoid dysplasia (asterisk) with atlantoaxial instability (arrow) and associated spinal stenosis as well as thoracolumbar kyphosis (double arrow) in MPS IVa patients also well delineated by CT images

In spine CT and plain radiographs, thoracic spinal abnormalities were grouped into two subtypes: subtype 1 (defined as thoracic kyphosis apex around T2, n = 8 or 66.67%) and subtype 2 (defined as thoracic kyphosis apex around T5, n = 4 or 33.33%) (Fig. 1; Table 1). However, one patient had a thoracic kyphosis apex around T1 to T2, while another patient’s kyphosis apex occurred at around T4 to T5 in subtypes 1 and 2, respectively. Square-shaped to mild central beaking in middle thoracic vertebral bodies was observed in subtype 1, whereas greater degrees of mild to moderate central beaking in middle thoracic vertebral bodies was observed in subtype 2 (Fig. 1; Table 1). In addition, we also delineated thoracolumbar kyphosis apex around T11 to L2, and the presence of craniocervical junction abnormalities as well as odontoid dysplasia in all twelve patients (Fig. 1). Lastly, the Cobb angle of thoracic kyphosis was measured, where subtype 2 cases seemed to have a greater Cobb angle of thoracic kyphosis compared to those in subtype 1 in our study (Table 1).

### Spine MRI findings

**Fig. 2:**
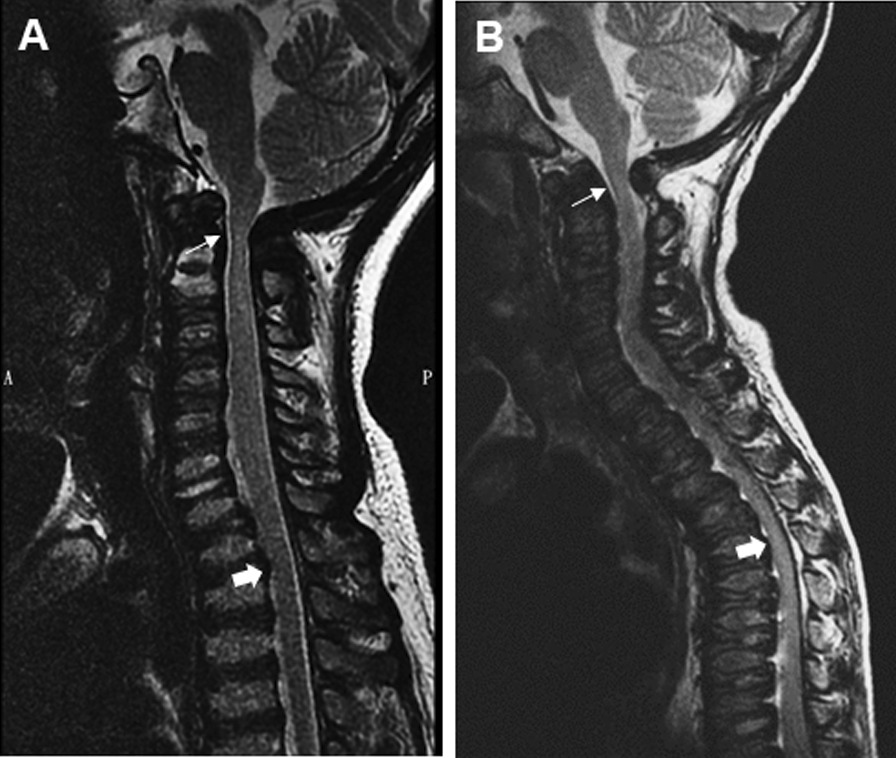
Spinal MRI images demonstration in MPS IVa patients. **A** MPS type IVa in a 14-year-old girl (subtype 1). Sagittal T2-weighted FSE MRI of the spine shows spinal canal narrowing around bony level of T2 (solid arrow) corresponding to CT manifestation of cervicothoracic kyphosis in addition to common spinal stenosis site around cervicomedullary junction (arrow). **B** MPS type IVa in a 5-year-old girl (subtype 2). T2-weighted FSE MRI of the spine in sagittal plane shows narrowing of the spinal canal more significant around bony level of T5 (solid arrow) related to CT reveals middle thoracic kyphosis. Atlantoaxial instability with mild cord myelopathy around cervicomedullary junction is also evident (arrow)

Spine MRI findings of the above eight MPS IVa patients showed relative narrowing of the spinal canal around the bony levels of T2 and T5 in subtypes 1 and 2, respectively (Fig. 2). Six patients with spinal stenosis at cervicomedullary junction was noted, three of which exhibited compressive myelopathy and received subsequent surgical decompression. However, no significant spinal cord compressive myelopathy was identified at thoracic and junction around thoracolumbar levels at the time of examination. Therefore, no surgical decompression at these levels was performed.

## Discussion

The present study may be the first to provide imaging evidence from CT and MRI of subtypes of thoracic spine morphology in MPS IVa patients. MPS-related spinal dysplasia develops in most MPS IVa patients but with varying severity. Results of the present study demonstrate abnormalities of thoracic spine dysplasia in patients with MPS IVa, including subtype 1 (defined as the thoracic kyphosis around T2) and subtype 2 (defined as the thoracic kyphosis around T5). In particular, consistency is noted between CT and MRI measurements in these patients. Overall, the combined radiological approach using CT and MRI still appears to be the best approach for screening full-spinal abnormalities in MPS IVa patients, and this pilot study has again demonstrated the importance of combined radiological monitoring for managing the clinical therapy of MPS IVa patients.

Typical spinal manifestations of MPS are atlantoaxial instability (with odontoid dysplasia), thoracolumbar kyphosis/scoliosis (gibbus deformity) and cervical/lumbar developmental spinal stenosis [12]. Spine disease and its severity depend on the type of MPS and may be associated with disease activity. Atlantoaxial instability has frequently been observed in MPS IV, followed by MPS VI and MPS I; thoracolumbar kyphosis is a well-known hallmark of MPS I, but is also common in MPS II, IV, and VI, and cervical stenosis is widespread in all types of MPS except MPS III [16, 17]. Similarly, the CT findings of the present study showed that craniocervical junction abnormalities and odontoid dysplasia were identified in all patients with MPS IVa. In addition, MPS IVa patients have common scoliosis and kyphosis of the thoracolumbar spine at T11 to L2 [18, 19], which was also observed in the present study. We further demonstrated that MPS IVa patients could be grouped into two subtypes, thoracic kyphosis apex around T2 (subtype 1) and thoracic kyphosis apex around T5 (subtype 2), although we also observed one patient in each subgroup with the thoracic kyphosis apex around T1/T2 and T4/T5 junction, which may be similar to results with a reported range of T11 to L2 instead of a precise bony level of the thoracolumbar apex. Meanwhile, the severity of central beaking in middle thoracic vertebral bodies and the Cobb angle of thoracic kyphosis were more significant in subtype 2 patients than in subtype 1 patients. Previous study has described a defect of vertebral body development causing thoracolumbar kyphosis [20]. Congenital cervical instability and kyphosis are the result of vertebral hypoplasia and are associated with a functional disconnection of the posterior spine associated with pedicle hypoplasia [21]. According to these findings, we speculated that the severity of central beaking in middle thoracic vertebral bodies might be a critical factor associated with thoracic kyphosis exhibiting an apex around different levels of T2 and T5 in MPS IVa patients. Certainly, it merits further investigation.

No guidelines are available currently for systematically assessing the extent of spinal involvement in patients with MPS IVa, as well as for identifying candidates for surgery or assessing the impact of treatment. Surgical decision making will be supported by the development of standardized risk stratification systems based on objective and measurable clinical, neurological, and radiological parameters. Although assessing dysplasia of the odontoid is more accurate in sagittal CT images than in MRI or plain radiographs due to easy identification of the lack of ossification, MRI clearly assesses a compressed spinal cord and is thus considered the gold standard for diagnosing spinal cord compression [22]. However, the incidence of spinal cord compression may be underestimated because MRI is usually performed in a neutral position. If the MRI option is available, it can also be used to assess the extent of compression [23]. Spinal cord compression occurs most commonly in two locations: the cervicomedullary junction and the thoracolumbar junction in children with thoracolumbar kyphosis [11]. Similarly, the spine MRI findings of the present study also indicated that compressed spinal cord around C1 to C2 could be clearly observed in the two subtypes of MPS IVa. Although we did not find severely compressed spinal cord around bony levels of T2, T5, and T11 to L2 in the two subtypes at the time of examination, results of previous studies indicated that evolution of vertebral abnormalities occurs over time in MPS patients [24, 25]. In a previous study, the kyphosis was progressive in MPS patients for whom serial radiographs were available [25], resulting in spinal problems requiring spinal decompression or fusion surgery [18]. Thus, long-term monitoring by MRI for spinal cord compression around T2 and T5 may be required in MPS IVa patients in addition to known common sites at the cervicomedullary and thoracolumbar junction. A scoring system based on MRI findings has been proposed for evaluating cervical spinal cord involvement in MPS patients [26], and a scoring system has been developed for determining the optimal timing of surgery for MPS IVa patients [27]. However, objective outcome measures and large well-designed studies are still needed to determine the efficacy of surgical and medical treatment for this patient population. Consequently, further investigation may be conducted to develop a modified scoring system for determining the optimal timing of surgery for the different subtypes of MPS IVa patients.

In a previous study where MPS-specific questionnaire was handed to patients, 68% of patients indicated that they had bone pain, and the most often reported joint pains were back pain (25.9%) and hip pain (27.8%) [28]. The pathophysiology of pain in patients with MPS is probably multifactorial. For example, glycosaminoglycan storage induces inflammation by activating toll-like receptor 4 and increasing levels of cytokines such as TNF-α has been shown with animal models in past literature [29, 30]. Furthermore, structural deformities of the spinal column may contribute to the onset of back pain, especially in the period of intense growth and development. Another previous study of MPS I patients reported cases with lumbar kyphosis had simultaneous low back pain [25]. In the present study, MPS IVa patients showed spinal kyphosis at bony levels of T2, T5, and T11 to L2. Further follow-up images are recommended for these patients if they have persistent or progressive back pain. Therefore, in addition to focusing on the changes in thoracolumbar junction that cause clinically relevant symptoms, we must also pay attention to the possible location of new thoracic spine abnormalities revealed in our study which will also cause related clinical symptoms.

The present study has several limitations. First, as this was a retrospective study, the potential for biases is inevitable. The study cohort was not large, and the study was conducted in a single medical center in Taiwan, which may limit the generalization of results to other centers, geographic locations or populations. As MPS IVa is considered an extremely rare disease, the sample size was quite limited and did not allow us to reach definitive conclusions. Another study or larger scale research are still needed to support our thoracic spinal image findings and correlate them with clinical presentation in various subtypes of MPS IVa patients.

## Conclusions

Spine CT findings clearly identify obvious bony abnormalities of cervical, thoracic, and lumbar spine in the subtypes of MPS IVa patients. MRI findings are consistent with CT measurements in these patients, and are important in neurological monitoring for spinal stenosis and the effects of clinical therapy. Overall, we suggest that the combined radiological approach using CT and MRI still provides the most appropriate and useful imaging techniques to screen for spinal abnormalities in various subtypes of MPS IVa patients. Our study results highlight again the unique image manifestations of thoracic kyphosis and associated spinal stenosis in MPS IVa patient population.

## Data Availability

The datasets used and/or analyzed during the current study are available from the corresponding author on reasonable request.

## Abbreviations

ADL: Activities of daily living
C6S: Chondroitin 6-sulphate
CT: Computed tomography
ERT: Enzyme replacement therapy
GAG: Glycosaminoglycans
GALNS: N-acetylgalactosamine-6-sulfate sulfatase
KS: Keratan sulphate
MPS: Mucopolysaccharidosis
MRI: Magnetic resonance imaging
